# Supplementary material for: Medication error reporting in Ghana: A multicenter assessment of healthcare professionals’ knowledge, attitudes and practices
Source: PLoS One. 2026 May 21;21(5):e0335116. doi: 10.1371/journal.pone.0335116 (PMC13193338; doi:10.1371/journal.pone.0335116)
Supplement: S1 File — (DOCX) [file pone.0335116.s001.docx]

| **Domain** | **Example Items** | **Response Options** | **Scoring / Interpretation** |
| --- | --- | --- | --- |
| **Knowledge** | *Do the following constitute categories of medication errors?* (e.g., wrong patient, wrong dose, wrong drug, wrong route, allergy-related error, wrong documentation) | Yes / No / Not sure | 1 = Correct; 0 = Incorrect or Not sure |
|  | *Do the following constitute sources of medication errors?* (e.g., prescribing, dispensing, administration errors, knowledge deficit, distractions, miscalculation) | Yes / No / Not sure | 1 = Correct; 0 = Incorrect or Not sure |
|  | *Do the following constitute contributory factors?* (e.g., negligence, lack of knowledge, communication failure, unclear orders, interruptions) | Yes / No / Not sure | 1 = Correct; 0 = Incorrect or Not sure |
| **Attitude / Awareness** | *Are you aware of a medication error reporting system in your facility?* | Yes / No | 1 = Yes; 0 = No |
|  | *Have you ever completed an incident reporting form?* | Yes / No | 1 = Yes; 0 = No |
|  | *Do you know how to locate/access the incident form?* | Yes / No | 1 = Yes; 0 = No |
|  | *Do you report errors using the incident/accident form?* | Always / Frequently / Half the time / Rarely / Never / No idea | 5–1 scale; higher = positive attitude towards reporting |
|  | *Do you know what to do with completed incident form?* | Yes / No | 1 = Yes; 0 = No |
| **Practice** | *Do you check patient identity before administering medication?* | Always / Frequently / About half / Rarely / Never | 5–1 scale; higher = better practice |
|  | *Do you prepare and carry medications for more than one patient at a time?* | Always / Frequently / About half / Rarely / Never | Reverse coded (1 = Always → 5 = Never) |
|  | *Do you label syringes and medication cups properly?* | Always / Frequently / About half / Rarely / Never | 5–1 scale; higher = better practice |
|  | *Do you have another clinician double-check high-risk medications (e.g., insulin)?* | Always / Frequently / About half / Rarely / Never | 5–1 scale; higher = better practice |

**Appendix 1**
