# Supplementary material for: Medication error reporting in Ghana: A multicenter assessment of healthcare professionals’ knowledge, attitudes and practices
Source: PLoS One. 2026 May 21;21(5):e0335116. doi: 10.1371/journal.pone.0335116 (PMC13193338; doi:10.1371/journal.pone.0335116)
Supplement: S2 File — (DOCX) [file pone.0335116.s002.docx]

**Supplementary Table S3:** Knowledge on categories and sources of medication errors

| **Variables** | **Yes n (%)** | **No n (%)** | **Not sure n (%)** |
| --- | --- | --- | --- |
| **Categories of Medication errors** |  |  |  |
| Wrong patient | 1680 (81.5) | 328 (15.9) | 54 (2.6) |
| Wrong dose | 1768 (85.5) | 263 (12.7) | 37 (1.8) |
| Wrong drug | 1698 (82.1) | 329 (15.9) | 41 (2.0) |
| Wrong route | 1645 (79.7) | 362 (17.5) | 58 (2.8) |
| Shortage of drug | 904 (44.0) | 966 (47.0) | 186 (9.0) |
| Wrong judgement | 1195 (59.0) | 620 (30.6) | 228 (11.3) |
| Wrong storage | 1327 (64.4) | 565 (27.4) | 167 (8.1) |
| Allergy-related error | 1230 (60.3) | 588 (28.8) | 222 (10.9) |
| Wrong documentation | 1467 (71.5) | 475 (23.2) | 109 (5.3) |
| No date/signature of order | 1240 (60.4) | 630 (30.7) | 183 (8.9) |
| Omission of drug | 1008 (48.9) | 769 (37.3) | 285 (13.8) |
| **Sources of medication errors** |  |  |  |
| Computer entry error | 1636 (79.7) | 243 (11.8) | 173 (8.4) |
| Transcription error | 1373 (66.8) | 409 (19.9) | 272 (13.2) |
| Order not received from pharmacy | 1066 (51.9) | 661 (32.2) | 326 (15.9) |
| Error checking medication sheet | 1371 (66.0) | 434 (20.9) | 250 (12.0) |
| Incorrect order | 1591 (77.2) | 336 (16.3) | 134 (6.5) |
| Confusing prescribing instructions | 1534 (74.9) | 321 (15.7) | 192 (9.4) |
| Misunderstood verbal order | 1586 (77.1) | 311 (15.1) | 160 (7.8) |
| Knowledge deficit (administration) | 1518 (73.8) | 367 (17.8) | 172 (8.4) |
| Distractions (administering) | 1436 (69.9) | 413 (20.1) | 206 (10.0) |
| Miscalculation | 1628 (79.0) | 325 (15.8) | 109 (5.3) |
| Procedure not followed (administering) | 1536 (74.7) | 363 (17.7) | 156 (7.6) |
| Filled incorrectly (dispensing) | 1536 (74.8) | 365 (17.8) | 152 (7.4) |
| Mislabeled (dispensing) | 1607 (78.1) | 342 (16.6) | 109 (5.3) |
| Drugs look alike | 1565 (76.0) | 329 (16.0) | 164 (8.0) |
| Labels look alike | 1581 (76.7) | 324 (15.7) | 155 (7.5) |

**Supplementary Table S4:** Individual and systems contributing factors that can contribute to medication errors

| **Variables** | **Yes n (%)** | **No n (%)** | **Not sure n (%)** |
| --- | --- | --- | --- |
| **Individual contributory factors** |  |  |  |
| Negligence/inattentiveness | 1729 (83.9) | 273 (13.3) | 58 (2.8) |
| Protocol not followed | 1676 (81.2) | 291 (14.1) | 96 (4.7) |
| Lack of knowledge | 1594 (77.4) | 375 (18.2) | 90 (4.4) |
| Practice beyond scope | 1294 (63.0) | 495 (24.1) | 266 (12.9) |
| Inappropriate communication | 1634 (79.2) | 348 (16.9) | 80 (3.9) |
| Drug abuse | 1312 (63.7) | 558 (27.1) | 190 (9.2) |
| Role overload | 1465 (72.2) | 350 (17.2) | 214 (10.5) |
| **Systems factors** |  |  |  |
| Inappropriate location | 1514 (73.8) | 365 (17.8) | 173 (8.4) |
| Unclear communication orders | 1621 (79.1) | 303 (14.8) | 126 (6.1) |
| No access to protocols | 1532 (74.7) | 365 (17.8) | 155 (7.6) |
| Interruption during preparation/admin | 1503 (74.7) | 357 (17.8) | 179 (7.6) |
| Inadequate technique/service | 1322 (64.6) | 450 (22.0) | 274 (13.4) |
| Pressure from patients/relatives/staff | 1334 (65.0) | 493 (24.0) | 224 (10.9) |
| Administration in emergency | 1295 (63.1) | 499 (24.3) | 258 (12.6) |
