# Supplementary material for: Medication error reporting in Ghana: A multicenter assessment of healthcare professionals’ knowledge, attitudes and practices
Source: PLoS One. 2026 May 21;21(5):e0335116. doi: 10.1371/journal.pone.0335116 (PMC13193338; doi:10.1371/journal.pone.0335116)
Supplement: Attachment — Submitted filename: REVIEW REPORT.pdf [file pone.0335116.s005.pdf]

REVIEW REPORT

SUMMARY OF THE MANUSCRIPT

This manuscript presents a large multicenter cross-sectional study (n=2,078) conducted across five teaching hospitals in Ghana, assessing healthcare professionals' knowledge, attitudes, and practices (KAP) relating to medication errors (MEs) and medication-error reporting systems. The authors examine knowledge of ME categories, adherence to key medication-administration safety practices, awareness and utilization of institutional reporting systems, and predictors of awareness (using Poisson regression).

The study is important and timely, given Ghana's growing focus on patient-safety systems, the WHO Global Patient Safety Action Plan, and documented underreporting of MEs across LMICs.

The manuscript is substantially improved from the tracked-changes version, but several scientific, methodological, clarity, and presentation issues remain. The comments below aim to further strengthen rigor, clarity, and alignment with PLOS ONE standards.

MAJOR COMMENTS

1. Internal Inconsistencies Between Clean Manuscript and Tracked-Changes Version
There are major inconsistencies between the clean manuscript and the tracked-changes version, particularly in:

- The regression analysis
- The interpretation of results
- The description of model choice
- Sections discussing specialty differences

For example:

- The clean manuscript presents Poisson regression with robust SEs (Table 4 in the clean version), consistent with the revised methods.
- The tracked-changes version still contains logistic regression outputs (AORs) and narrative explanations contradicting the Poisson model.
- Two different tables for "factors associated with awareness" appear (pages 20–22), with conflicting interpretations.

Recommendation:
A final reconciliation is required. Remove all outdated regression tables and text from the tracked-changes manuscript and ensure one final, consistent version is submitted.

2. Rationale for Poisson Regression Should Be Better Justified
The clean manuscript states that because the outcome prevalence is >10%, Poisson regression with robust SEs is appropriate. This is correct, but the manuscript should further justify model selection, including:

- Confirmation that variance inflation or overdispersion was tested.
- Clarification of whether multicollinearity was assessed (VIF or similar).
- Clear specification of all covariates included in the model.

Recommendation:
Consider adding a short paragraph in Methods - Data Analysis describing:

- diagnostic tests performed
- model-fit checks (e.g., goodness-of-fit, multicollinearity assessment)

This will strengthen statistical transparency.

3. Missing Conceptual Link Between Knowledge, Practice, and Reporting Behaviour
The manuscript presents excellent descriptive statistics, but lacks an integrated conceptual narrative. For instance:

- Knowledge levels were high for traditional ME categories, but low for system-level MEs.
- Unsafe practices (preparing medications for >1 patient at a time, administering medications prepared by others) were common.
- Reporting awareness and utilization were low.

However, the manuscript does not connect these observations into an integrated explanation of why reporting behaviour is poor.
Recommendation:
Strengthen Discussion by explicitly linking:

- Knowledge gaps, unsafe practices, reporting barriers, system weaknesses
- How this aligns with Reason's Swiss Cheese Model or Vincent's London Protocol

Right now, the London Protocol is mentioned but could be used more meaningfully to interpret Ghana-specific systemic barriers.

4. Interpretation of Results Still Contains Causal Language
Although the revision attempted to address this, some causal phrasing remains.
Examples include:

- "These practice gaps may reflect workplace pressures…" (suggests causality)
- "This underreporting limits the healthcare system's ability…"
- "The finding that more experienced staff showed lower awareness suggests that…"

These are borderline causal for a cross-sectional study.
Recommendation:
Use non-causal language such as *"may be associated with"*, *"is consistent with"*, or *"could potentially indicate."*

5. Tables Are Dense and Could Be Streamlined
Tables 2 and 3 contain extremely long lists of variables, many of which convey the same directional interpretation.
Recommendation:
For publication readability:

- Move detailed lists to Supplementary Material.
- Keep summary/aggregated categories in the main manuscript (e.g., high-knowledge, moderate-knowledge, low-knowledge clusters).
- Provide a simplified figure summarizing KAP patterns.

6. Figures Need Clearer Titles and Labels

- Figure 1: Adherence to precautions
  The bars are visible, but the y-axis labelling ("Percentage (%)") and the interpretation of colours need clearer explanation.
- Figure 2: Reporting system awareness and practices
  Should explicitly label each panel and indicate sample sizes.

Recommendation:
Provide fully descriptive figure captions explaining what each sub-figure represents.

7. Measurement Validity Needs More Detail
Although the authors mention content validity and CVI >0.80, important aspects remain unclear:

- Were the knowledge items adapted from existing validated tools (e.g., NCC MERP taxonomy)?
- How were "correct answers" determined for knowledge items?
- Were internal consistency metrics (e.g., Cronbach alpha) calculated?

Recommendation:
Add reliability assessment (Cronbach α) for knowledge, practice, and attitude scales.

8. Discussion Should Address the Very High Participation Rate
Response rates of 82–87% are impressive. The authors should discuss:

- How recruitment was conducted (ward-based vs. supervisory selection)
- Whether participation was voluntary and free from coercion
- Whether non-response bias is plausible

This is relevant for PLOS ONE's commitment to ethical transparency.

9. Table and Figure Headings Lack Appropriate Statistical Descriptions
Throughout the manuscript, the titles and headings of tables and figures do not explicitly state the statistical content, which is required for clarity and alignment with PLOS ONE's reporting standards.
Examples:

- Table 1 presents frequencies and percentages, but the heading does not state *"Frequency distribution"* or *"Descriptive statistics"*.
- Tables 2 and 3 provide item-level frequencies but lack descriptors such as *"Distribution of responses (%)"* or *"Knowledge item frequency distribution"*.
- The Poisson regression table (Table 4) does not explicitly state:
    - the model type
    - outcome variable
    - statistical metric (e.g., prevalence ratios with 95% CI)

Similarly:

- Figure 1 (medication administration precautions) lacks a clear statistical label such as *"Distribution of self-reported practice frequencies (%)"*.
- Figure 2 (reporting system awareness) needs clarification that it shows *"Proportion of healthcare workers responding to reporting-system awareness and utilization items"*.

Recommendation:
Every table and figure should include, in both title and caption:

- the type of statistic presented (frequency, percentage, prevalence ratio, etc.),
- the model used, where applicable (e.g., Poisson regression with robust standard errors),
- the population denominator,
- a brief explanation of how the variables were measured.

This will greatly improve transparency and scientific rigor.

10. No Defined Benchmarks for Categorizing Knowledge, Attitudes, and Practices
The manuscript describes findings such as:

- "Knowledge was high for wrong-dose errors"
- "Knowledge was moderate for documentation-related errors"
- "Knowledge of drug-shortage errors was low"

However, no explicit scoring framework or benchmark criteria were established to define:

- What constitutes *high knowledge*
- What constitutes *moderate knowledge*
- What constitutes *low knowledge*
- Whether these are based on absolute percentages or predefined thresholds

This creates interpretive ambiguity—readers cannot independently reproduce or validate these categories.

Recommendation:

The authors should define and justify interpretive cut-offs, for example:

- High knowledge: ≥75% correct
- Moderate knowledge: 50–74% correct
- Low knowledge: <50% correct

OR justify alternative cut-offs using:

- literature benchmarks
- psychometric distribution characteristics
- expert consensus

These benchmarks should be clearly stated in the Methods → Measurement subsection and referenced in the Results.

Without this, interpretation of “high”, “moderate”, and “low” is subjective and unverifiable.

MINOR COMMENTS

1. Headings Within Tables Should Use Correct Statistical Terms

Examples requiring revision:

- “Variables”, “Variables and Response Frequencies (%)”
- “Factors associated with awareness”, “Poisson Regression Model of Factors Associated with Awareness (Prevalence Ratios and 95% CI)”

2. Ensure Consistency in Numbering of Tables and Figures

Some tables appear twice or with inconsistent numbering between clean and tracked versions.

3. Clarify Denominator Variability in a Table Note

As noted earlier, denominators change across tables. Add:

“Denominators differ due to item non-response.”

4. Remove Residual Editorial Marks

Some markup comments (e.g., “Formatted: Font…”) still appear in the tracked version. These must be deleted entirely.

OVERALL RECOMMENDATION

Major Revision

The manuscript remains strong and highly publishable, but the newly identified issues—especially the lack of statistical descriptors in table headings, absence of interpretive benchmarks, and formatting inconsistencies, must be addressed before final acceptance.
