# Supplementary material for: Medication error reporting in Ghana: A multicenter assessment of healthcare professionals’ knowledge, attitudes and practices
Source: PLoS One. 2026 May 21;21(5):e0335116. doi: 10.1371/journal.pone.0335116 (PMC13193338; doi:10.1371/journal.pone.0335116)
Supplement: Attachment — Submitted filename: PLOS_ONE_Reviewer_Report_PONE-D-25-54066.docx [file pone.0335116.s003.docx]

# PLOS ONE Reviewer Report

Manuscript Title: Medication Error Reporting in Ghana: A Multicenter Assessment of Healthcare Professionals’ Knowledge, Attitudes and Practices

Manuscript ID: PONE-D-25-54066

## Reviewer Comments to Authors

### 1. Summary

This study examines healthcare professionals’ knowledge, attitudes, and practices regarding medication-error reporting in five Ghanaian teaching hospitals (n = 2,078). Using a validated questionnaire and descriptive plus logistic analyses, the authors identify high theoretical awareness but low utilization of reporting systems. The work addresses a crucial yet under-researched patient-safety issue in low- and middle-income countries and aligns well with WHO’s Medication Without Harm initiative.

### 2. General Assessment

The manuscript is well organized and clearly written. It contributes valuable multicenter evidence from Ghana, but it requires methodological clarification, analytic transparency, and deeper theoretical contextualization before it can meet PLOS ONE standards of reproducibility and interpretive rigor.

Overall recommendation: **Major revision**

### 3. Major Comments

**Sampling and representativeness:** Clarify how sampling ensured representativeness within each hospital and report participation/response rates and handling of missing data.

**Questionnaire details:** Provide an appendix or supplementary table showing key items, response options, and scoring for the Knowledge–Attitude–Practice domains. It is not explicitly clear, for example, which responses constitute attitudes and practices.

**Statistical modeling:** Indicate dependent variable coding, independent variables entered, confounders adjusted for, and model-fit indices such as Hosmer–Lemeshow and pseudo-R².

**Interpretation of associations:** Acknowledge the cross-sectional design limitation and avoid causal phrasing such as 'led to' or 'resulted in'.

**Discussion depth:** Integrate Reason’s Swiss Cheese model and/or Vincent’s London Protocol to interpret system and cultural barriers to reporting.

**Policy linkage:** Discuss how findings could inform Ghana Health Service or Ministry of Health policies on non-punitive reporting and continuing professional development.

**Data sharing compliance:** Provide an anonymized dataset or repository link to satisfy PLOS ONE data-availability policy.

### 4. Minor Comments

Replace 'Data was analyzed' with 'Data were analyzed'.
Correct 'responders will t feel' to 'respondents will feel'.
Use 'Obstetrics and Gynecology (O&G)' instead of 'O & G/delivery suits'.
Maintain consistent spelling of behavior.
Ensure uniform capitalization of 'Knowledge, Attitudes, and Practices'.
Verify numerical consistency in tables, as some denominators differ from 2,078.
Simplify dense tables and consider adding a visual summary such as a bar chart of adherence or awareness rates.

### 5. Strengths to Retain

Large and diverse multicenter dataset.
Robust ethical governance and validation process.
Timely relevance to LMIC patient-safety agendas.
Clear and well-written results section with helpful tables.

### 6. Summary of Required Revisions

1. Expand methodological transparency regarding sampling, instrument, and regression.
2. Deepen discussion with systems-safety frameworks.
3. Improve clarity, grammar, and table consistency.
4. Upload dataset or repository link.
5. Check reference and format compliance with PLOS ONE style.

When these revisions are addressed, the manuscript will make a significant contribution to global medication-safety literature.

## Confidential Comments to the Editor

This is a well-executed multicenter KAP study of medication-error reporting among Ghanaian healthcare professionals—a field with limited empirical data from Sub-Saharan Africa. The sample size of about 2,000 participants and coverage across five teaching hospitals lend credibility and external validity. However, the paper currently reads more as a descriptive audit than an analytical or theory-driven study. Statistical transparency and interpretive depth must be improved before acceptance. The methodology appears sound, and no ethical or scientific integrity concerns are noted. If the authors adequately address the methodological clarifications and enrich the discussion, this manuscript would be suitable for publication. I recommend major revision at this stage.
