# Supplementary material for: Medication error reporting in Ghana: A multicenter assessment of healthcare professionals’ knowledge, attitudes and practices
Source: PLoS One. 2026 May 21;21(5):e0335116. doi: 10.1371/journal.pone.0335116 (PMC13193338; doi:10.1371/journal.pone.0335116)
Supplement: Attachment — Submitted filename: Response to reviewers comments.docx [file pone.0335116.s006.docx]

**Response to reviewer’s comments**

**Reviewer #1:**

**Results**
**Comment:** It is observed that there is no mention of the tabulated results in the result report. Authors are advised to address this so that readers can follow what tabulated results is being reported.
**response:** Thank you for your observation. The suggested corrections have been made in the revised manuscript.

**Comment: Line 327:** Authors have to look at that sentence and put it in a way that conveys what they intend to put out. The sentence does not make sense.

**Response:** The sentence has been revised in the new manuscript to ensure clarity

Comment: The study is very relevant, and it present a critical health practise concern. However, it would be essential to show or demonstrate the work relationship across the health professionals of these facilities. Was there a case of inferiority complex setting in, or intimidation or outright negligence and lack of facility members not getting upgraded with best drug reporting and administration practices? It would be also essential to probe into if the facilities carry out routine trainings to update the staff on recent practices. The drawback is observed with having the newer staff having the knowledge and skills to report while the older or more experienced members are lagging behind. Such submission is a possible indictment on the facility.

**Response:** Thank you for these insightful observations. We fully agree that interpersonal dynamics among healthcare professionals, potential hierarchical influences, and the role of routine training are important factors that could affect medication error reporting and patient safety culture.

The scope of the present study, however, was limited to quantitatively assessing awareness and associated factors related to medication error reporting systems. The dataset did not include items that directly measured interpersonal relationships, intimidation, hierarchical culture, or institutional training practices; therefore, we were unable to empirically evaluate these aspects within the current analysis.

These issues indeed represent important complementary dimensions of medication safety and reporting behavior. They form part of a broader arm of our ongoing research programme, which will involve qualitative methods (e.g., in-depth interviews and focus groups) to explore staff experience, communication patterns, teamwork climate, facility culture, and perceived barriers such as intimidation or lack of support.

We have now added a statement in the revised manuscript acknowledging this limitation and highlighting the need for qualitative and mixed-methods studies to better understand system-level and cultural factors influencing medication error reporting in Ghanaian hospitals. We believe this addition strengthens the manuscript and aligns with the reviewer’s suggestion.

We appreciate the reviewer’s concern regarding the interpretation that the difference in reporting between newer and older staff could be seen as indicting and a reflection on the facility’s performance. To prevent any misinterpretation or controversy, we have carefully rephrased the discussion following our revised analysis as requested by the other reviewer.

**Reviewer #2**

**Comment: Sampling and representativeness:** Clarify how sampling ensured representativeness within each hospital and report participation/response rates and handling of missing data.

**Response:** We appreciate the reviewer’s request for clarification on sampling and representativeness. These suggestions have been included in the revised manuscript to improve transparency and allow readers to assess the generalizability of our findings (page 6, lines 110-113, and page 8, lines 146-152).

**Comment: Questionnaire details:** Provide an appendix or supplementary table showing key items, response options, and scoring for the Knowledge–Attitude–Practice domains. It is not explicitly clear, for example, which responses constitute attitudes and practices.

**Response:** Thank you for this insightful comment. We appreciate the reviewer’s suggestion to provide greater clarity on the structure and scoring of the questionnaire. In response, we have now added Appendix 1 to the manuscript. The appendix presents the key items grouped under the Knowledge, Attitude, and Practice domains, together with their response options and scoring systems. In addition, we have updated the Methods section (page 7, line 121-128) as follows:

*“The structured questionnaire comprised three sections: (i) knowledge of medication errors (categories, sources, and contributory factors), (ii) attitudes and awareness towards reporting medication errors, and (iii) medication administration practices. Knowledge items were scored 1 for correct and 0 for incorrect or ‘not sure’ responses. Attitude items were scored on a dichotomous (Yes/No) or Likert-type scale, where higher scores indicated positive attitudes towards reporting. Practice items were rated on a five-point frequency scale (1 = never to 5 = always), with higher scores indicating safer medication practices. The classification of items and scoring criteria are illustrated in Appendix 1.”*

**Comment: Statistical modeling:** Indicate dependent variable coding, independent variables entered, confounders adjusted for, and model-fit indices such as Hosmer–Lemeshow and pseudo-R².

**Response:** Thank you for this helpful comment. We initially conducted binary logistic regression analysis as indicated in the original submission. However, upon re-examination following the reviewer’s feedback, we found that the model diagnostics (including the Hosmer–Lemeshow test and pseudo-R² values) indicated poor model fit and potential overestimation of associations due to the relatively high prevalence of the outcome variable (awareness of medication error reporting system).

Consequently, we re-analyzed the data using Poisson regression with robust standard errors to estimate prevalence ratios (PRs) instead of odds ratios (ORs). This approach is statistically more appropriate when the outcome is common (>10%) and provides a more interpretable measure of association.

The revised results, presented in the updated Table 6, now report prevalence ratios with 95% confidence intervals. We have also updated the *Statistical Analysis* subsection in the Methods to reflect this change. This adjustment improves both the validity and interpretability of the findings while maintaining transparency with the review process.

We believe this revision directly addresses the reviewer’s concern regarding model specification and strengthens the analytical rigor of the study

**Comment: Interpretation of associations:** Acknowledge the cross-sectional design limitation and avoid causal phrasing such as 'led to' or 'resulted in'.

**Response:** Thank you for this helpful comment. We agree with the reviewer that the cross-sectional design limits our ability to infer causality. We have carefully revised the manuscript to avoid any causal language such as *‘led to’*, *‘caused’*, or *‘resulted in’*, and have replaced them with non-causal terms such as *‘associated with’*, *‘linked to’*, or *‘related to’*.

**Discussion depth:** Integrate Reason’s Swiss Cheese model and/or Vincent’s London Protocol to interpret system and cultural barriers to reporting.

**Response:** Thank you for this insightful suggestion. We agree that situating our findings within a patient-safety framework enhances the theoretical depth and interpretive value of the discussion. Accordingly, we have revised the discussion section to integrate Vincent’s London Protocol in interpreting the system-level factors and barriers that influence medication error reporting in Ghanaian healthcare settings.

**Comment: Policy linkage:** Discuss how findings could inform Ghana Health Service or Ministry of Health policies on non-punitive reporting and continuing professional development.

**Response:** Thank you for this insightful comment. We agree that linking the study’s findings to national health policy frameworks will enhance the practical relevance of our work. Accordingly, we have expanded the Discussion and Conclusion sections to highlight how the results can inform Ghana Health Service (GHS) and Ministry of Health (MoH) policies (page 28, lines 408-420; and page 29, lines 445-446)

**Comment:** **Data sharing compliance:** Provide an anonymized dataset or repository link to satisfy PLOS ONE data-availability policy.

**Response:** data supporting this study has been included as supplementary information

**Minor Comments**

Replace 'Data was analyzed' with 'Data were analyzed'.
Correct 'responders will t feel' to 'respondents will feel'.
Use 'Obstetrics and Gynecology (O&G)' instead of 'O & G/delivery suits'.
Maintain consistent spelling of behavior.
Ensure uniform capitalization of 'Knowledge, Attitudes, and Practices'.
Verify numerical consistency in tables, as some denominators differ from 2,078.
Simplify dense tables and consider adding a visual summary such as a bar chart of adherence or awareness rates.

**Responses:** We are grateful to the reviewer for such valuable insights. Minor grammatical errors that were pointed out have been corrected in the revised manuscript as suggested by the reviewer. However, concerning the issue of, “some denominators differ from 2,078,” we must emphasize that final analysis of the data took into consideration the number of participants who responded to those items, hence the difference in denominators for the various demographic categories.
