# Supplementary material for: Medication error reporting in Ghana: A multicenter assessment of healthcare professionals’ knowledge, attitudes and practices
Source: PLoS One. 2026 May 21;21(5):e0335116. doi: 10.1371/journal.pone.0335116 (PMC13193338; doi:10.1371/journal.pone.0335116)
Supplement: Attachment — Submitted filename: Recommendation to the Editor.docx [file pone.0335116.s007.docx]

**Recommendation to the Editor**

Following a careful assessment of the revised manuscript (R2) and the authors’ detailed responses to my review comments, I am satisfied that the authors have adequately addressed all substantive methodological, analytical, and conceptual concerns raised in the previous review round.

The manuscript has improved markedly in terms of:

- Statistical appropriateness and transparency, with consistent use of Poisson regression and clearer justification of model choice;
- Internal consistency, with reconciliation of earlier discrepancies between manuscript versions;
- Conceptual integration, particularly in linking knowledge, practices, and reporting behaviour within a systems and patient-safety framework;
- Presentation and clarity, including improved tables, figures, and narrative flow.

The few remaining issues are purely editorial or stylistic in nature (e.g., minor refinements to table headings, formatting consistency) and can be readily addressed during copy-editing. They do not affect the scientific validity, robustness, or interpretability of the findings.

**Overall Recommendation**

**Accept for Publication**

I recommend that the manuscript be accepted for publication in *PLOS ONE*. The study represents a valuable and well-executed contribution to the literature on medication safety and error reporting in low- and middle-income settings, and it is well aligned with the journal’s scope and standards.
