# Supplementary material for: Medication error reporting in Ghana: A multicenter assessment of healthcare professionals’ knowledge, attitudes and practices
Source: PLoS One. 2026 May 21;21(5):e0335116. doi: 10.1371/journal.pone.0335116 (PMC13193338; doi:10.1371/journal.pone.0335116)
Supplement: Attachment — Submitted filename: Response to editor Comments.docx [file pone.0335116.s009.docx]

**Response to editor Comments**

**Comment:** In Table 4, the authors presented only adjusted prevalence ratio without presenting the crude. It is recommended that the authors present both the crude and adjusted and also explain what informed the variable selection for the multivariable model.

**Response:** We thank the editor for this important comment. In response, we have revised Table 4 to include both crude prevalence ratios (cPR) and adjusted prevalence ratios (aPR) with 95% confidence intervals and p-values. Regarding variable selection, all exposure variables were included simultaneously in the multivariable Poisson regression model based on theoretical relevance and prior literature, rather than on statistical significance alone. This approach was chosen to account for potential confounding and to estimate the independent effect of each variable while controlling for others. We have clarified this in the Methods section.
