# Supplementary material for: Medication error reporting in Ghana: A multicenter assessment of healthcare professionals’ knowledge, attitudes and practices
Source: PLoS One. 2026 May 21;21(5):e0335116. doi: 10.1371/journal.pone.0335116 (PMC13193338; doi:10.1371/journal.pone.0335116)
Supplement: Attachment — Submitted filename: Response to Review Comment.docx [file pone.0335116.s010.docx]

**Response to Review Comment**

**Additional Editor Comments
Comment 1.** There should be consistency in the reporting of p-values. Eg. in Table 4, some of the p-values are in 1, 2 and 3 decimal places. All should be consistent throughout the manuscript

**Response:** Thank you for this observation. We have revised Table 4 and the entire manuscript to ensure consistent reporting of p-values.

**Comment 2.** Subsequently, all exposure variables were included together in a single multivariate model, so that the effect of each variable was estimated while accounting for the others. Delete the word 'multivariate' from this sentence as authors are not assessing more than one outcome

**Response:** Thank you for this observation. We agree that the analysis involved a single outcome; therefore, the term multivariate has been removed.

**Comment 3.** Provide a reference for this statement. This approach was preferred over logistic regression because the outcome on awareness was common (>10) and PRs provide a more interpretable measure of association. All exposure variables were included in the multivariable model based on theoretical relevance and prior evidence.

**Response:** Thank you for this helpful comment: Appropriate references have been provided to support the statements in the revised manuscript.

**Comment 4.** Multicollinearity among predictors was assessed using variance inflation factors (VIFs); the mean VIF was 1.27, indicating no evidence of problematic multicollinearity. Report the minimum and maximum values for the variables in the model for the VIF in addition to the mean VIF.

**Response:** Thank you for this suggestion. We have now revised the Methods Results section to include the range of variance inflation factors. The VIF values in our study ranged from 1.01 to 1.66, with a mean of 1.27, indicating no evidence of problematic multicollinearity.
