# Supplementary material for: Medication error reporting in Ghana: A multicenter assessment of healthcare professionals’ knowledge, attitudes and practices
Source: PLoS One. 2026 May 21;21(5):e0335116. doi: 10.1371/journal.pone.0335116 (PMC13193338; doi:10.1371/journal.pone.0335116)
Supplement: Attachment — Submitted filename: Response to review Comments.docx [file pone.0335116.s011.docx]

**Response to review Comments**

**Additional editor comments**

**Comment:** I appreciate the authors for responding to the comments that were raised in my last review. However, not all of them have been addressed. Especially, Table 4 still has some of the p-values presented in 3 decimals while some also are presented in 2. Variable "Age (9yrs)" is an error and should be corrected by the authors. Again, 'Age' in all the tables should be changed to "Age group (years)". The results write-up also need to be re-written. The current format report almost everything in the Tables and should not be so. The Table is there as a reference and authors should not report everything in it in the write up.

**Response:** Thank you for the careful evaluation of the manuscript. We have addressed the remaining concerns as follows: (1) p-values in Table 4 and throughout the manuscript have been standardized to ensure consistent decimal formatting; (2) the variable previously labeled “Age (9yrs)” has been corrected, and “Age” has been revised to “Age group (years)”; and (3) the Results section has been revised to reduce repetition of numerical values already presented in the tables, with the text now summarizing the key findings while the tables provide the detailed data. These revisions have been incorporated into the manuscript.
