# Supplementary material for: Medication error reporting in Ghana: A multicenter assessment of healthcare professionals’ knowledge, attitudes and practices
Source: PLoS One. 2026 May 21;21(5):e0335116. doi: 10.1371/journal.pone.0335116 (PMC13193338; doi:10.1371/journal.pone.0335116)
Supplement: Attachment — Submitted filename: Response_to_editor_comments_auresp_6.docx [file pone.0335116.s012.docx]

**Response to review comments**

**Comment:** The results write up still needs to be improved. Add the estimates in bracket. Eg majority (23.3%) of the participants were males etc.

Response: Thank you for this helpful comment. The results section has been revised to include the percentages in brackets. In addition, totals have been removed from Table 1 as recommended to improve presentation.
