# Supplementary material for: Medication error reporting in Ghana: A multicenter assessment of healthcare professionals’ knowledge, attitudes and practices
Source: PLoS One. 2026 May 21;21(5):e0335116. doi: 10.1371/journal.pone.0335116 (PMC13193338; doi:10.1371/journal.pone.0335116)
Supplement: Attachment — Submitted filename: Response_to_editor_comments_auresp_7.docx [file pone.0335116.s013.docx]

**Response to editor comments**

**Comment:** Please take your time and address the concerns I raised in my last review. You only addressed the concerns I raised partially. Apart from removing the total from Table 1, the other comment was not addressed. Re-write your results section and do not state everything in the table. Also just mentioning majority without stating the percentage is not enough.

**Response:** Thank you for your detailed feedback on our manuscript. I appreciate the time you have taken to review our work and provide constructive comments. The manuscript has been revised accordingly. The Results section has been re-written to avoid repetition of table content and to provide clearer interpretation of key findings. In addition, all descriptive terms such as “majority” are now supported with corresponding percentages.
