# Supplementary material for: Medication error reporting in Ghana: A multicenter assessment of healthcare professionals’ knowledge, attitudes and practices
Source: PLoS One. 2026 May 21;21(5):e0335116. doi: 10.1371/journal.pone.0335116 (PMC13193338; doi:10.1371/journal.pone.0335116)
Supplement: Attachment — Submitted filename: Response to reviewer comments.docx [file pone.0335116.s008.docx]

Response to reviewer comments

**Comment** 1. Internal Inconsistencies Between Clean Manuscript and Tracked-Changes Version

There are major inconsistencies between the clean manuscript and the tracked-changes version, particularly in:

- The regression analysis
- The interpretation of results
- The description of model choice
- Sections discussing specialty differences

For example:

- The clean manuscript presents Poisson regression with robust SEs (Table 4 in the clean version), consistent with the revised methods.
- The tracked-changes version still contains logistic regression outputs (AORs) and narrative explanations contradicting the Poisson model.
- Two different tables for “factors associated with awareness” appear (pages 20–22), with conflicting interpretations.

Recommendation: A final reconciliation is required. Remove all outdated regression tables and text from the tracked-changes manuscript and ensure one final, consistent version is submitted.

**Response:** Thank you for these important observations. The tables/descriptions that seemed to appear twice in pages 20-22, only showed the changes that were made to the initial manuscript. The initial table with AORs, and its descriptions were all edited following the reviewer’s suggestion, as evidenced by the crossed lines through them. It only appears to show what was actually removed/changes that were made. All the necessary changes have been made to in the revised version of the manuscript.

**Comment 2:** Rationale for Poisson Regression Should Be Better Justified

The clean manuscript states that because the outcome prevalence is >10%, Poisson regression with robust SEs is appropriate. This is correct, but the manuscript should further justify model selection, including:

- Confirmation that variance inflation or overdispersion was tested.
- Clarification of whether multicollinearity was assessed (VIF or similar).
- Clear specification of all covariates included in the model.

Recommendation: Consider adding a short paragraph in Methods - Data Analysis describing:

- diagnostic tests performed
- model-fit checks (e.g., goodness-of-fit, multicollinearity assessment)

This will strengthen statistical transparency.

**Response:** We thank the reviewer for this suggestion. Multicollinearity was assessed using variance inflation factors, and the mean VIF was 1.27, indicating no evidence of problematic multicollinearity. This information has now been included in the Data Analysis section of the Methods. We have also explicitly listed all covariates included in the multivariable Poisson regression model in the footnote of Table 4 to improve transparency and interpretability. This has now been explicitly stated in the Methods (data analysis section).

**Comment 3**. Missing Conceptual Link Between Knowledge, Practice, and Reporting Behaviour

The manuscript presents excellent descriptive statistics but lacks an integrated conceptual narrative. For instance:

- Knowledge levels were high for traditional ME categories, but low for system-level MEs.
- Unsafe practices (preparing medications for >1 patient at a time, administering medications prepared by others) were common.
- Reporting awareness and utilization were low.

However, the manuscript does not connect these observations into an integrated explanation of why reporting behaviour is poor.

Recommendation: Strengthen Discussion by explicitly linking:

- Knowledge gaps, unsafe practices, reporting barriers, system weaknesses
- How this aligns with Reason’s Swiss Cheese Model or Vincent’s London Protocol

Right now, the London Protocol is mentioned but could be used more meaningfully to interpret Ghana-specific systemic barriers.

**Response:** We thank the reviewer for highlighting the need for a clearer conceptual linkage between knowledge, practice, and reporting behavior. In response, we have added a new paragraph in the Discussion describing the Conceptual Integration of Knowledge, Practice, and Reporting Behavior, in which we explicitly interpret our findings using Vincent’s London Protocol and Reason’s Swiss Cheese Model, with attention to Ghana-specific system-level barriers.

**Comment 4:** Interpretation of Results Still Contains Causal Language

Although the revision attempted to address this, some causal phrasing remains.

Examples include:

- “These practice gaps may reflect workplace pressures…” (suggests causality)
- “This underreporting limits the healthcare system’s ability…”
- “The finding that more experienced staff showed lower awareness suggests that…”

These are borderline causal for a cross-sectional study.

Recommendation: Use non-causal language such as *“may be associated with”*, *“is consistent with”*, or *“could potentially indicate.”*

**Response:** Thank you for these insightful observations. All causal phrases have been removed from the revised manuscript.

**Comment 5**: Tables Are Dense and Could Be Streamlined

Tables 2 and 3 contain extremely long lists of variables, many of which convey the same directional interpretation.

Recommendation: For publication readability:

- Move detailed lists to Supplementary Material.
- Keep summary/aggregated categories in the main manuscript (e.g., high-knowledge, moderate-knowledge, low-knowledge clusters).
- Provide a simplified figure summarizing KAP patterns.

**Response:** We thank the reviewer for this insightful comment. In response, we have moved the detailed item-level list of medication error categories and sources to the Supplementary Material (Supplementary Tables S3 and S4). In the main manuscript, we now present aggregated knowledge categories (high, moderate, and low knowledge) to improve clarity and readability. Additionally, we have included a simplified figure (Figure 1) summarizing overall knowledge patterns among healthcare professionals.

**Comment 6:** Figures Need Clearer Titles and Labels

- Figure 1: Adherence to precautions The bars are visible, but the y-axis labelling (“Percentage (%)”) and the interpretation of colours need clearer explanation.
- Figure 2: Reporting system awareness and practices Should explicitly label each panel and indicate sample sizes.

Recommendation: Provide fully descriptive figure captions explaining what each sub-figure represents.

**Response:** We thank the reviewer for this helpful comment. The figures have been revised to include clearer titles, explicit y-axis labelling (“Percentage of respondents (%)”), and fully descriptive annotations. Figure 2 has been updated to clearly label each panel (A–E) for more clarity. These revisions ensure that all figures are self-explanatory and interpretable as standalone elements.

**Comment 7:** Measurement Validity Needs More Detail

Although the authors mention content validity and CVI >0.80, important aspects remain unclear:

- Were the knowledge items adapted from existing validated tools (e.g., NCC MERP taxonomy)?
- How were “correct answers” determined for knowledge items?
- Were internal consistency metrics (e.g., Cronbach alpha) calculated?

Recommendation: Add reliability assessment (Cronbach α) for knowledge, practice, and attitude scales.

**Responses:** We thank the reviewer for highlighting the need for additional detail on measurement validity and reliability. We have revised the Methods section to clarify that the questionnaire was informed by established patient safety frameworks, including the NCC MERP taxonomy, and adapted to the local clinical context. We have also clarified how correct responses for knowledge items were defined and scored. In addition, we have now included internal consistency reliability assessments, with Cronbach’s alpha values reported for the knowledge, attitude, and practice subscales.

**Comment 8**. Discussion Should Address the Very High Participation Rate

Response rates of 82–87% are impressive. The authors should discuss:

- How recruitment was conducted (ward-based vs. supervisory selection)
- Whether participation was voluntary and free from coercion
- Whether non-response bias is plausible

This is relevant for PLOS ONE’s commitment to ethical transparency

**Response:** We thank the reviewer for this observation. We have revised the Methods section to clarify that participants were recruited using a ward-based approach, with voluntary participation and no supervisory involvement, thereby minimizing the risk of coercion. In the Discussion, we now explicitly address the high participation rate, its potential explanations, and the likelihood of non-response bias, in line with PLOS ONE’s emphasis on ethical transparency.

**Comment 9**. Table and Figure Headings Lack Appropriate Statistical Descriptions

Throughout the manuscript, the titles and headings of tables and figures do not explicitly state the statistical content, which is required for clarity and alignment with PLOS ONE’s reporting standards.

Examples:

- Table 1 presents frequencies and percentages, but the heading does not state *“Frequency distribution”* or *“Descriptive statistics”*.
- Tables 2 and 3 provide item-level frequencies but lack descriptors such as *“Distribution of responses (%)”* or *“Knowledge item frequency distribution”*.
  The Poisson regression table (Table 4) does not explicitly state: the model type
  outcome variable
  statistical metric (e.g., prevalence ratios with 95% CI)

Similarly:

- Figure 1 (medication administration precautions) lacks a clear statistical label such as *“Distribution of self-reported practice frequencies (%)”*.
- Figure 2 (reporting system awareness) needs clarification that it shows *“Proportion of healthcare workers responding to reporting-system awareness and utilization items”*.

Recommendation: Every table and figure should include, in both title and caption:

- the type of statistic presented (frequency, percentage, prevalence ratio, etc.),
- the model used, where applicable (e.g., Poisson regression with robust standard errors),
- the population denominator,
- a brief explanation of how the variables were measured.

This will greatly improve transparency and scientific rigor.

**Response:** We thank the reviewer for this valuable recommendation. In response, we have revised all table titles and figure captions to explicitly state the type of statistic presented, the analytical model used where applicable, the population denominator, and a brief description of how variables were measured. These revisions ensure that all tables and figures are fully self-explanatory and enhance the transparency and scientific rigor of the manuscript in line with PLOS ONE reporting standards.

**Comment: 10.** No Defined Benchmarks for Categorizing Knowledge, Attitudes, and Practices

The manuscript describes findings such as:

- “Knowledge was high for wrong-dose errors”
- “Knowledge was moderate for documentation-related errors”
- “Knowledge of drug-shortage errors was low”

However, no explicit scoring framework or benchmark criteria were established to define:

- What constitutes *high knowledge*
- What constitutes *moderate knowledge*
- What constitutes *low knowledge*
- Whether these are based on absolute percentages or predefined thresholds

This creates interpretive ambiguity—readers cannot independently reproduce or validate these categories.

Recommendation: The authors should define and justify interpretive cut-offs, for example:

- High knowledge: ≥75% correct
- Moderate knowledge: 50–74% correct
- Low knowledge: <50% correct

OR justify alternative cut-offs using:

- literature benchmarks
- psychometric distribution characteristics
- expert consensus

These benchmarks should be clearly stated in the Methods → Measurement subsection and referenced in the Results.

Without this, interpretation of “high”, “moderate”, and “low” is subjective and unverifiable.

**Response:** We thank the reviewer for this important comment. We agree that explicit benchmarks are necessary to avoid interpretive ambiguity. We have now defined and justified percentage-based thresholds for interpreting item-level knowledge, with ≥75% correct responses classified as high knowledge, 50–74% as moderate knowledge, and <50% as low knowledge. These cut-offs have been clearly stated in the Methods section and referenced in the Results to ensure transparency and reproducibility.

MINOR COMMENTS

1. Headings Within Tables Should Use Correct Statistical Terms

Examples requiring revision:

- “Variables”, “Variables and Response Frequencies (%)”
- “Factors associated with awareness”, “Poisson Regression Model of Factors Associated with Awareness (Prevalence Ratios and 95% CI)”

2. Ensure Consistency in Numbering of Tables and Figures

Some tables appear twice or with inconsistent numbering between clean and tracked versions.

3. Clarify Denominator Variability in a Table Note

As noted earlier, denominators change across tables. Add:

*“Denominators differ due to item non-response.”*

4. Remove Residual Editorial Marks

Some markup comments (e.g., “Formatted: Font…”) still appear in the tracked version. These must be deleted entirely.

**Response:** We thank the reviewer for these helpful suggestions. Table headings have been revised to use appropriate statistical terminology, including explicit references to frequency distributions and Poisson regression models with prevalence ratios and 95% confidence intervals. Table and figure numbering has been standardized across all manuscript versions. A clarifying note has been added to indicate that denominators vary due to item non-response. Finally, all residual editorial and formatting marks have been removed from the manuscript.
